# Pilot Survey of Breast Cancer Management in Sub-Saharan Africa

**DOI:** 10.1200/JGO.2016.004945

**Published:** 2016-12-21

**Authors:** Verna D.N.K. Vanderpuye, Olufunmilayo I. Olopade, Dezheng Huo

**Affiliations:** **Verna D.N.K. Vanderpuye**, Korlebu Teaching Hospital, Accra, Ghana; and **Olufunmilayo I**. Olopade and **Dezheng Huo**, University of Chicago, Chicago, IL

## Abstract

**Purpose:**

To understand the current state of breast cancer management in sub-Saharan Africa.

**Methods:**

We conducted an anonymous online survey of breast cancer management among African Organization for Research and Treatment in Cancer (AORTIC) members by using a 42-question structured questionnaire in both English and French in 2013.

**Results:**

Twenty members from 19 facilities in 14 countries responded to the survey. Twelve members (60%) belonged to a multidisciplinary breast cancer team. Radiotherapy equipment was available in seven facilities (36%), but equipment had down time at least once a week in four facilities. Available chemotherapy drugs included methotrexate, cyclophosphamide, fluorouracil, anthracyclines, and vincristine, whereas trastuzumab, taxanes, vinorelbine, and gemcitabine were available in few facilities. Core-needle biopsy was available in 16 facilities (84%); mammogram, in 17 facilities (89%); computed tomography scan, in 15 facilities (79%); magnetic resonance imaging, in 11 facilities (58%); and bone scans, in nine facilities (47%). It took an average of 1 to 3 weeks to report histopathology. Immunohistochemistry was available locally in eight facilities (42%), outside hospitals but within the country in seven facilities (37%), and outside the country in four facilities (21%). Thirteen facilities (68%) performed axillary node dissections as part of a breast protocol. Neoadjuvant chemotherapy was the most common therapy for locally advanced breast cancer in 13 facilities (68%). In three facilities (16%), receptor status did not influence the prescription of hormone treatment.

**Conclusion:**

This pilot survey suggests that AORTIC members in sub-Saharan Africa continue to make gains in the provision of access to multidisciplinary breast cancer care, but the lack of adequate pathology and radiotherapy services is a barrier. Focused attention on in-country and regional training needs and improvement of health systems deliverables is urgently needed.

## INTRODUCTION

Breast cancer is the most common cancer in women worldwide, and at least 50% of breast cancer occurrences and 62% of deaths occur in less-developed countries.^1^ This pattern is attributed to limited availability of screening, late presentation, and poor access to treatment, which includes surgery, chemotherapy, and radiation therapy. The availability of such treatments in sub-Saharan Africa is limited because of lack of skilled manpower, surgical equipment, and radiation facilities. According to a review in 2010 from the International Atomic Energy Agency, 29 of 52 countries in Africa have no radiation treatment facilities; more than half of the existing machines are located in southern and northern Africa.^2^ Pathology services, in general, are lower than the recommended standards required for adequate management decision making. Delays in reporting of test results invariably affect local control and survival outcomes. Multidisciplinary tumor boards are mandatory in most institutions worldwide, but the practicality of operating tumor boards in sub-Saharan Africa is limited by the lack of physicians with oncology skills and the existence of few tertiary institutions that can organize tumor boards on a consistent basis. Comparatively, however, better-performing economies located in the southern and northern parts of Africa have well-equipped centers and governmental support, which leaves the sub-Saharan region in dire need of cancer support services.

Every African country should begin to make investments to deal with the impending cancer burden as a result of the aging population and the adoption of sedentary lifestyle, increased alcohol and tobacco consumption, and underdeveloped primary preventive strategies. To address this growing cancer burden, the African Organization for Research and Training in Cancer (AORTIC) has been committed to fostering research, education, and advocacy on several levels to increase awareness of cancer in Africa. To understand the current state of breast cancer management in sub-Saharan Africa, we conducted a pilot online survey by using the AORTIC membership directory, which included 35 countries in Africa.

## METHODS

An anonymous and voluntary online survey of facilities for breast cancer management was conducted among AORTIC members from 31 sub-Saharan African countries who practiced in Africa with direct patient contact by using a 42-question structured survey in both English and French. Sections of the questionnaire included the following: (1) availability of skilled manpower, (2) availability and function of radiotherapy equipment, (3) availability and cost of chemotherapy drugs and other medications, (4) diagnosis and general management of breast cancer, and (5) suggestions to improve health care delivery. The results were compiled with Microsoft Excel (Microsoft, Redmond, WA) and analyzed with STATA (STATA, College Station, TX). Results are presented in frequency tables and graphs, as appropriate. The survey received exempt status by the institutional review boards at the University of Chicago (Chicago, IL) and the Korlebu Teaching Hospital (Accra, Ghana).

## RESULTS

Twenty members from 19 facilities in 14 countries responded to the online survey. Most respondents were oncologists, which included six clinical oncologists (31.6%), three surgical oncologists (15.8%), two radiation oncologists (10.5%), and one medical oncologist (5.3%). There were two general surgeons (10.5%) and five other health care professionals, which included oncology nurses, palliative care nurses, surgical pathologists, and public health specialists (Table 1). Twelve institutions (60%) had a breast multidisciplinary team.

**Table 1:**
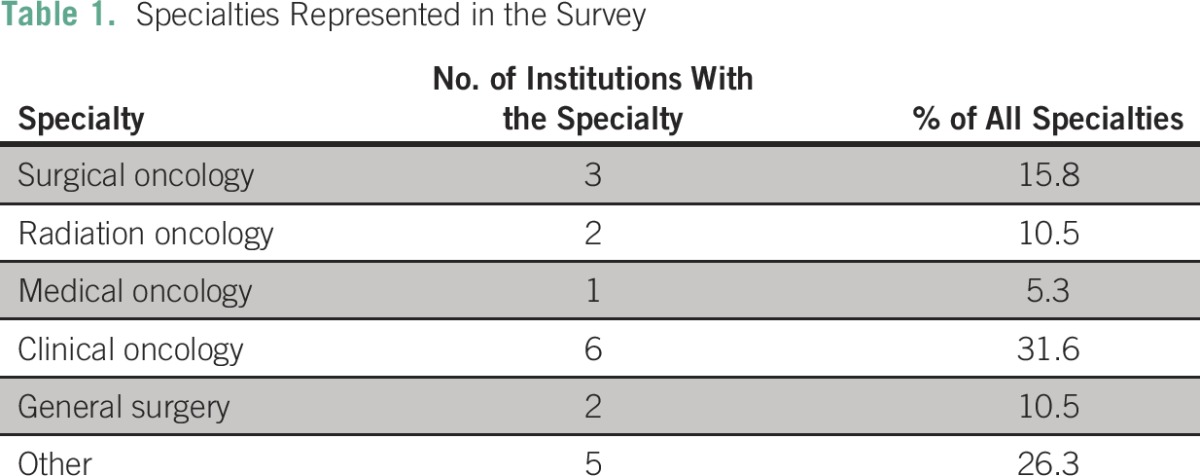
Specialties Represented in the Survey

### Radiotherapy Equipment

Eleven facilities (58%) had no radiotherapy equipment. Five centers (26%) had both a Cobalt 60 teletherapy machine and a linear accelerator, two centers (11%) had only a Cobalt 60 machine, and one center (5%) had only a linear accelerator (Table 2). Half of these available machines (50%) broke down less than once a week.

**Table 2:**
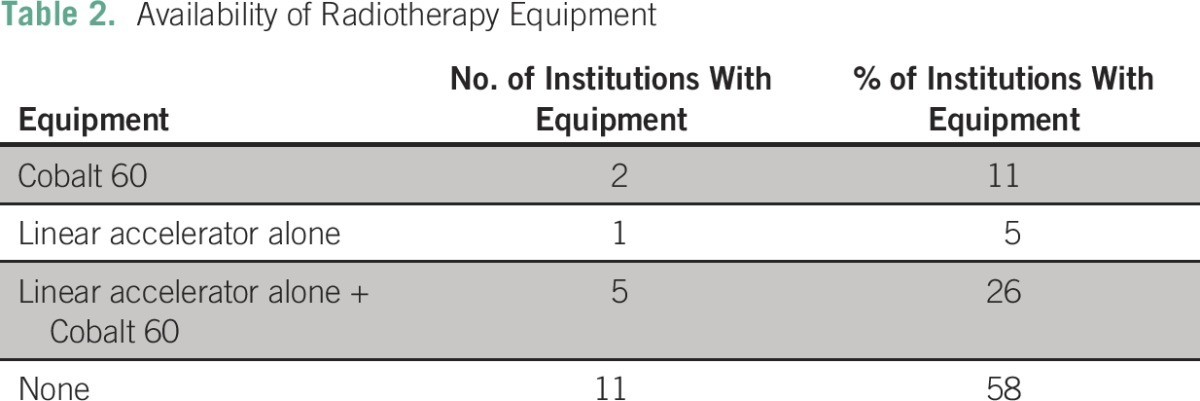
Availability of Radiotherapy Equipment

### Pathology Services

Sixteen facilities (85%) confirmed diagnoses of breast cancer by using a core-needle biopsy technique. Pathology services were available locally within the country in 15 facilities (79%). However, half of these services were based outside of the facility. Four facilities (21%) accessed pathology services in neighboring countries. Nine facilities (47.8%) had immunohistochemistry services within their laboratories, seven (36.8%) had these services outside the laboratory but within the country, and three facilities (15.8%) had these services outside of the country. The average turnaround time for pathology reports was 1 to 3 weeks. Pathology reports in all facilities included size of tumor; total number of lymph nodes removed; and number positive for disease, grade, margin status, and presence of lymph vascular invasion.

### Imaging Capabilities

Availability of radiologic equipment is summarized in Table 3; a high percentage of institutions had computed tomography scans, mammography, and ultrasonography. Bone scintigraphy was present in less than half of institutions, and magnetic resonance imaging was present in 58%.

**Table 3:**
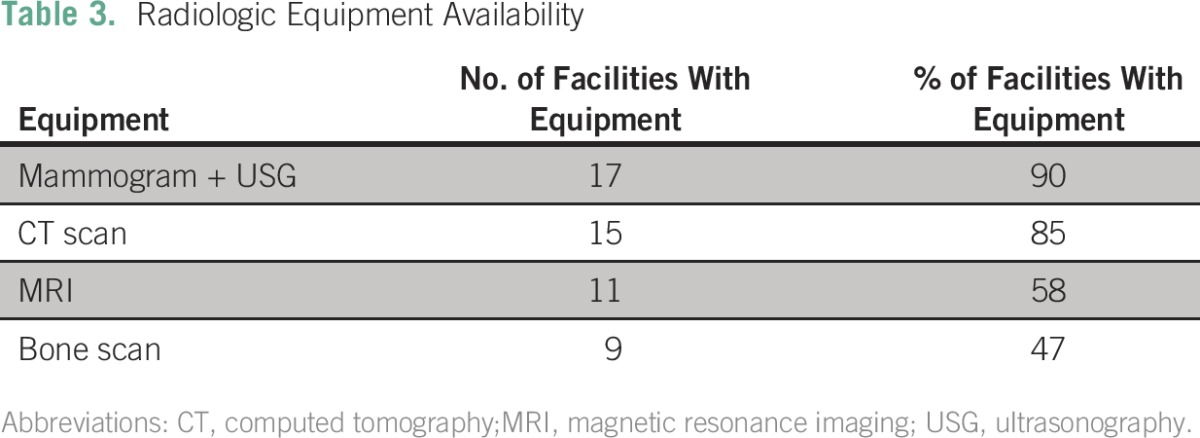
Radiologic Equipment Availability

### Management Practices

Facilities reported that 40% to 90% of their patients presented initially with locally advanced or metastatic disease. Twelve facilities (60%) participated in tumor boards. Surgery is the initial treatment of choice for early-stage disease, and 67% of facilities performed regular axillary lymph node dissections. The median number of lymph nodes removed was six (range, 0 to 15); more than 70% of facilities harvested at least six nodes. Only three facilities (15%) harvested more than nine nodes.

When available, radiotherapy began within 4 to 6 weeks of completion of the last chemotherapy. Neoadjuvant chemotherapy was frequently recommended for locally advanced disease in 13 facilities (67%). Knowledge of hormone receptor status affected treatment decision in the majority of cases (75%), whereas a few facilities did not rely on hormone receptor status to make treatment decisions. First-line chemotherapy in the majority of facilities (60%) consisted of anthracycline-based chemotherapy. Taxanes were recommended in a few instances, especially for triple-negative breast cancer. Second-line therapies included taxanes, vinorelbine, and gemcitabine, irrespective of hormone receptor status.

### Availability of Chemotherapy and Hormonal Therapy

**Fig 1:**
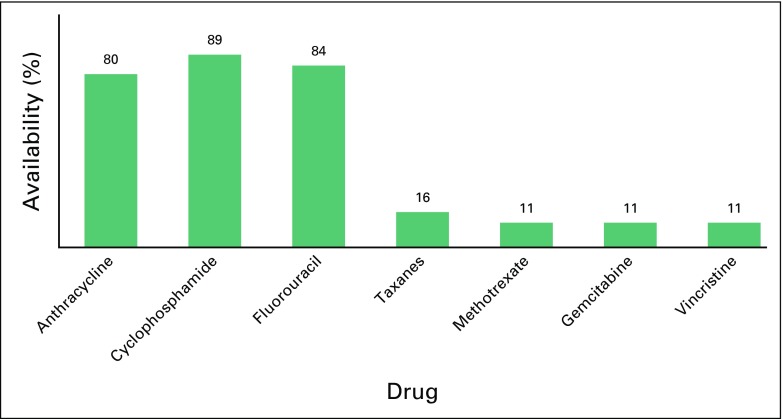
Availability of chemotherapy drugs (% of facilities).

**Fig 2:**
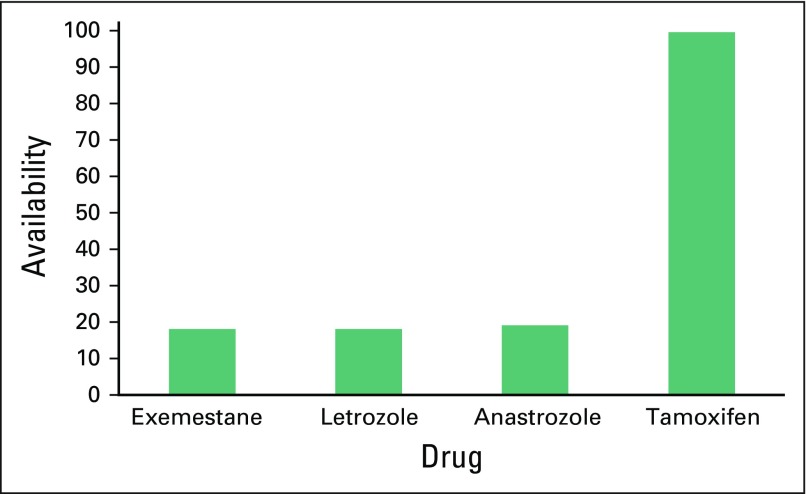
Availability of hormonal therapies for breast cancer management (no. of facilities).

With the exception of trastuzumab, generic antineoplastic drugs were highly preferred in all institutions (100%). Ten (50%) facilities had trastuzumab available, but less than 5% of patients could afford it. Half of these facilities used the 9-week FinHer schedule of nine weekly doses of trastuzumab and docetaxel followed by three cycles of cyclophosfamide, epirubicin, and flourouracil.^3^ Notably, the recommended 1-year schedule for trastuzumab was preferred mainly in the southern part of Africa. Greater than 80% of institutions used chemotherapy combinations of doxorubicin, cyclophosphamide, and fluorouracil. Less than 20% of institutions had access to taxanes, methotrexate, gemcitabine, or vinorelbine (Fig 1). Tamoxifen was readily available in all institutions, whereas aromatase inhibitors were only available in less than 20% of facilities (Fig 2).

### Factors That Contribute to Poor Outcome

Respondents offered opinions about factors that contribute to poor outcomes of breast cancer. Advanced stage at presentation (75%), financial constraints (52%), cultural practices (48%), and logistics (44%) were considered important barriers to care.

## DISCUSSION

Breast cancer is an important health concern for women in Africa; more than half of patients die as a result of the disease compared with less than a quarter in developed countries. Most low- to middle-income countries (LMICs), including those of sub-Saharan Africa, report high death rates as a result of advanced-stage breast cancer of 50% to 70%.^4^ Multifactorial contributors for the high case-fatality rate include poverty; sociocultural conflicts; late diagnoses and referral patterns among health personnel; lack of oncology training in surgical, pathologic, and medical fields; and poor access to few available resources.

Africa constitutes a wide spectrum of economies; the northern sector and South Africa are relatively better equipped with cancer treatment capabilities and comprehensive national health insurance schemes. Assessment of access and availability of cancer care in Africa tends to overestimate resources. Therefore, a separate assessment of the sub-Saharan region will reflect the true state of affairs. This survey was done through AORTIC to shed light on the management of breast cancer in sub-Saharan Africa and to bring to light progress made as well as limitations and areas for attention.

There were several limitations to this study. It surveyed only small numbers of countries and respondents, it captured only centers with some form of oncologic services, and it only included members of AORTIC.

Advanced stage at presentation is perceived to be the most common cause of poor treatment outcomes for breast cancer, because it increases cost and also reduces the available treatment options. Respondents reported that 40% to 90% of patients present with advanced disease, and this maybe a consequence of delay in diagnoses and treatments. In a study from Taiwan,^5^ a delay in seeking treatment of more than 2 months resulted in a high recurrence rate. Patients who received treatment had a 5-year overall survival of 85% versus 45% for those who did not receive or had delayed treatment. These delays were attributed to sociocultural practices, use of alternative therapies, and physician-related issues—none of which are unusual to sub-Saharan Africa.

Financial constraint is a major cause of delay in seeking medical attention. This is because most LMICs with low health budgets have large out-of-pocket payments for direct and indirect medical expenses related to cancer care, which patients and families consider unaffordable.^6^ Apart from out-of-pocket payments, logistical constraints in the health care facilities also play a major part in delaying treatment because of long waiting times for breast cancer surgery and radiotherapy, if they are available at all.

With respect to availability of diagnostic methods, it is highly commendable that members prefer core-needle biopsies for diagnostic purposes, because it improves the accuracy of diagnosis. For many years, fine-needle aspiration has been the hallmark of diagnosis and has had sensitivity rates of 84% to 98% and specificity rates of more than 99%. Although it is cost effective, fine-needle aspiration frequently needs to be repeated, whereas core-needle biopsies allow more diagnostic and prognostic information to be obtained and have an accuracy of 98.2%, a sensitivity of 95%, and a specificity of 100%.^7,8^ AORTIC has provided the platform to improve the pathologic services among its members by organizing several workshops and research projects across the continent. Immunohistochemistry to determine the receptor status of breast cancer specimens is not easily and readily accessible to facilities. The need for such services to improve management decisions and outcomes, however, is recognized among members, which is leading to outsourcing of this test if it is locally unavailable. This test is considered a standard part of the assessment of cancer. Stringent quality assurance testing must be used in the local laboratories to ensure reproducibility of test results. This is necessary, because accuracy of hormone receptor, human epidermal growth factor receptor 2, and Ki67 status is required to guide appropriate management.

This survey shows that most facilities have adequate imaging services except for bone scintigraphy. Less than half of respondents had access to bone scans, which may lead to suboptimal staging and management. The cumbersome processes and high cost to import radioactive material that supports bone scintigraphy may be limiting factors.

Respondents were mostly clinical and surgical oncologists. Clinical oncologists practice both radiation and medical oncology, which is a practice less common in the developed world. Rapid advances in radiation oncology and the increasingly complex nature of medical management of tumors underscore the justification for the separate specialization of medical and radiation oncologist. The role of the clinical oncologist is still important in Africa, where there is a paucity of human resources and there are limited oncology training facilities.

Cancer surgeries are performed frequently by general surgeons in Africa, but few centers provide structured training in surgical oncology. Sullivan et al^9^ recently evaluated the quality of cancer surgery in low-resource settings and concluded that a lot of work is required to improve the quality of cancer surgery and that there especially is a need to address the severe shortfall of cancer surgeons. Countries and regions need to make urgent efforts to build local capacity for training and retain these highly skilled physicians, which will in effect meaningfully influence treatment outcomes.

The role of physicians trained in oncology to direct and discuss patient management is paramount to the improvement of treatment. Breast cancer treatments managed by tumor board decisions generally have better outcomes, and a tumor board improves the management skills of the team.^10^ Ideally, every new patient with breast cancer should have their case discussed at a multidisciplinary tumor board before any interventions. Sixty percent of facilities have established tumor boards to discuss disease management for individual patients, which indicates a commitment to improve patient care.

It was observed that radiotherapy machines were not available to most respondents. Africa has only 2% of the world’s radiotherapy machines, of which most are in the northern and southern parts of Africa.^11^ An International Atomic Energy Agency report^12^ indicated that Egypt and Morocco had greater than 85% coverage of the population by radiotherapy equipment and that South Africa had 100% coverage but had problems with accessibility, especially for the rural poor, whereas Libya had at least 70% coverage but lacked the requisite skilled personnel to make it widely accessible. Comparatively, in Uganda, only 5% of patients who required radiation received it. Breast conservation is only possible when radiation treatments are available. In sub-Saharan Africa, the few women who present with early disease will undergo mastectomies and lose their breasts, and the majority who present with locally advanced disease will have high rates of local recurrence because of the limited access to radiotherapy. Poorly negotiated maintenance agreements and the low quality of electricity supply lead to frequent breakdowns and, subsequently, to inefficient treatments. Many African countries have acquired sophisticated radiation delivery systems that include complex linear accelerators, but those countries may lack the required advanced technological competence, from maintenance to operation, to ensure proper applications. The Cobalt 60 teletherapy machine was the choice for LMICs because of its lower initial cost, reliability, and simplicity of operation. However, challenges with replacement of the spent source and the high cost of source disposal have reduced its overall cost effectiveness. A majority of patients in the region present with advanced or metastatic disease, so palliative radiation therapy is an important tool necessary for symptom control. Therefore, governments in sub-Saharan Africa need to commit more to obtain and maintain radiotherapy facilities and need to invest in the training and maintenance of skilled personnel.

Modified radical mastectomy is the most common surgical procedure performed and could be attributed to the increased number of patients who present with advanced disease. The lack of and unreliability of radiation facilities in institutions preclude the number of breast-conserving surgeries performed. The majority of institutions performed axillary node dissections as a surgical routine, but the number of lymph nodes routinely harvested varied: less than 20% harvested more than nine nodes, and 70% harvested at least six nodes. Neoadjuvant chemotherapy is the most common chemotherapy prescribed and is related to the high number of advanced-stage disease.

The survey showed that the most available chemotherapy schedule is cyclophosphamide, doxorubicin, and fluorouracil, which indicates that the most basic and effective chemotherapy combination according to the WHO essential drug list is available to most patients. Conversely, taxanes used sequentially with cyclophosphamide, doxorubicin, and fluorouracil for advanced disease are not readily available and are used more frequently in subsequent-line settings. The choice to incorporate a taxane is determined by disease characteristics, cost, and choice of treating physician or availability. Trastuzumab was rarely affordable, because generic forms are unavailable and inaccessible. The standard of practice for *HER2/neu*-positive breast cancer is 1 year of adjuvant trastuzumab with chemotherapy.^13^ However, shorter regimens that consist of 9 weeks of trastuzumab with chemotherapy have been reported from Finland and are considered highly cost effective.^14^ This protocol is being adopted by most African physicians because of cost constraints. A randomized trial to compare 1 year of versus 9 weeks of trastuzumab treatment is long overdue and may answer questions about noninferiority. Tamoxifen was readily available compared with aromatase inhibitors across all facilities. At least the most basic form of hormonal therapy applicable across all ages of patients with hormone receptor–positive breast cancer was accessible across the subregion. Notably, a few respondents did not base management decisions on hormone receptor status. This raises concerns, because there are no survival benefits when patients with hormone receptor–negative breast cancer receive hormonal therapies^15^; also, patients with the low-risk hormone receptor–positive breast cancer subtype (luminal A) derive little benefit from adjuvant chemotherapy and may be unnecessarily treated with expensive and toxic chemotherapy regimens in the absence of testing.^16^ It is expected that, over time, some current management practices will change with wider accessibility to evidence-based resource-appropriate treatment guidelines, improved human resources in oncology, and participation in tumor board discussions.

All respondents in the survey highly patronized generic anticancer drugs to ensure affordability and compliance to treatment. The cost of generic cytotoxic drugs is estimated to save 70% to 80% off of the cost of patented drugs and is considered a cost-effective option for lower-income countries.^17^ Issues raised are clinical bioequivalence and lack of stringent measures in countries to prevent the influx of substandard and counterfeit drugs. Patients with incurable diseases could have improved qualities of life and sometimes better survival with anticancer medication; thus, the inaccessibility of anticancer drugs should be considered a grave injustice to the women in sub-Saharan Africa.

The economic implications of a diagnosis of breast cancer can be devastating for families and could be an important contributing factor to late presentation, which is estimated to be as high as 70%. With the low gross domestic product and the high cost of cancer therapies in LMICs, 32% to 42% on average of the household income might go toward out-of-pocket medical bills for patients with cancer.^18^ The resulting financial burden trickles down to affect the health of other family members, because there are limited funds available to cover the cost of other ailments. In a small study from Haiti, despite free treatments, 40% of patient income was eroded by out-of-pocket indirect medical costs.^19^ The average per capita health expenditure for African countries is approximately 82 dollars, and it is estimated that 50% to 70% of the health care cost is from private funds.^20^ Cancer competes with other diseases, including malaria, HIV, and tuberculosis, in the health budget allocations for African countries. A lot of good will is required from governments to increase spending on cancer-related issues.

Civil societies, including nongovernmental organizations, that deal with breast cancer need to do more to sensitize politicians to improve health coverage for the most common cancer in women. Sociocultural influences and poverty promote the behavior of seeking alternative treatments from traditional healers and priests, because their solutions are considered cheaper and less invasive, have the promise of limited adverse effects, and transcend beyond the physical realm.

In conclusion, AORTIC members promote multidisciplinary tumor boards, improved histopathology reporting, and immunohistochemistry testing for appropriate breast cancer care. Hindrances to optimal breast cancer management include the following: lack of reliable radiotherapy facilities and anticancer medications; lack of skilled health workers across the oncology spectrum; lack of political will to tackle the cancer burden with adequate financial commitments; and lack of research interest in cancer-related areas. Regional training of a skilled oncology workforce and improvement of health care delivery are needed to improve the quality of breast cancer treatment in Africa. A follow-up survey will be conducted to evaluate changes and improvements in breast cancer management resources in sub-Saharan Africa.

## References

[1] Torre LA, Bray F, Siegel RL (2015). Global cancer statistics, 2012. CA Cancer J Clin.

[2] Atun R, Jaffray DA, Barton MB (2015). Expanding global access to radiotherapy. Lancet Oncol.

[3] Joensuu H, Kellokumpu-Lehtinen PL, Bono P (2006). Adjuvant docetaxel or vinorelbine with or without trastuzumab for breast cancer. N Engl J Med.

[4] Brinton LA, Figueroa JD, Awuah B (2014). Breast cancer in sub-Saharan Africa: Opportunities for prevention. Breast Cancer Res Treat.

[5] Chen SJ, Kung P-T, Huang KH (2015). Characteristics of the delayed or refusal therapy in breast cancer patients: A longitudinal population-based study in Taiwan. PLoS One.

[6] Vanderpuye VDNK, Yarney J (2014). Cost effectiveness of cancer therapies in Africa. J Med Diagn Meth.

[7] Rikabi A, Hussain S (2013). Diagnostic usefulness of tru-cut biopsy in the diagnosis of breast lesions. Oman Med J.

[8] Homesh NA, Issa MA, EL-Sofiani HA. The diagnostic accuracy of fine needle aspiration cytology versus core needle biopsy for palpable breast lumps. Saudi Med J 26:42-46, 2005 15756351

[9] Sullivan R, Alatise OI, Anderson BO (2015). Global cancer surgery: Delivering safe, affordable, and timely cancer surgery. Lancet Oncol.

[10] El Saghir NS, Charara RN, Kreidieh FY, et al: Global Practice and Efficiency of Multidisciplinary Tumor Boards: Results of an American Society of Clinical Oncology international survey. J Global Oncol 1:57-64, 2015 10.1200/JGO.2015.000158 PMC5539869 28804774

[11] Samiei M: Challenges of making radiotherapy accessible in developing countries, in Magrath I (ed): Cancer Control 2012: Cancer Care Emerging Health Systems. Brussels, Belgium, International Network for Cancer Treatment and Research, 2012, pp 87-99

[12] Abdel-Wahab M, Bourque JM, Pynda Y, et al: Status of radiotherapy resources in Africa: An International Atomic Energy Agency analysis. Lancet Oncol 14:e168-e175, 2013 10.1016/S1470-2045(12)70532-6 23561748

[13] Goldhirsch A, Gelber RD, Piccart-Gebhart MJ (2013). 2 years versus 1 year of adjuvant trastuzumab for HER2-positive breast cancer (HERA): An open-label, randomised controlled trial. Lancet.

[14] Purmonen TT, Pänkäläinen E, Turunen JHO (2011). Short-course adjuvant trastuzumab therapy in early stage breast cancer in Finland: Cost-effectiveness and value of information analysis based on the 5-year follow-up results of the FinHer trial. Acta Oncol.

[15] Swain SM: Tamoxifen for patients with estrogen receptor–negative breast cancer. J Clin Oncol 19:93s-97s, 2001 (suppl) 11560981

[16] Uchida N, Suda T, Ishiguro K (2013). Effect of chemotherapy for luminal a breast cancer. Yonago Acta Med.

[17] Lopes Gde L: Cost comparison and economic implications of commonly used originator and generic chemotherapy drugs in India. Ann Oncol 24:v13-v16, 2013 (suppl 5) 10.1093/annonc/mdt323 23975699

[18] Mahal A, Karan A, Fan VY, et al: The economic burden of cancers on indian households. PloS One 8:e71853, 2013 10.1371/journal.pone.0071853 PMC3741186 23951258

[19] O’Neill KM, Mandigo M, Pyda J (2015). Out-of-pocket expenses incurred by patients obtaining free breast cancer care in Haiti: A pilot study. Surgery.

[20] Stefan DC: Cancer care in Africa: An overview of resources. J Global Oncol1:30-36, 2015 10.1200/JGO.2015.000406 PMC5551648 28804769

